# Comparative analysis of complete chloroplast genome sequences of four major *Amorphophallus* species

**DOI:** 10.1038/s41598-018-37456-z

**Published:** 2019-01-28

**Authors:** Erxi Liu, Chaozhu Yang, Jiangdong Liu, Surong Jin, Nunung Harijati, Zhongli Hu, Ying Diao, Lingling Zhao

**Affiliations:** 1 State Key Laboratory of Hybrid Rice, Lotus Engineering Research Center of Hubei Province, College of Life Science, Wuhan University, Wuhan, Hubei 430072 P. R. China; 2 Institute of Konjac, Enshi Academy of Agricultural Sciences, Enshi, P. R. China; 3 School of Chemistry, Chemical Engineering and Life Science, Wuhan University of Technology, Wuhan, 430070 P. R. China; 4 Department of Biology, Faculty of Mathematics and Natural Sciences, Brawijaya University, Jl. Veteran, Malang, 65145 Indonesia

## Abstract

*Amorphophallus* (Araceae) contains more than 170 species that are mainly distributed in Asia and Africa. Because the bulbs of *Amorphophallus* are rich in glucomannan, they have been widely used in food, medicine, the chemical industry and so on. To better understand the evolutionary relationships and mutation patterns in the chloroplast genome of *Amorphophallus*, the complete chloroplast genomes of four species were sequenced. The chloroplast genome sequences of *A*. *albus*, *A*. *bulbifer*, *A*. *konjac* and *A*. *muelleri* ranged from 162,853 bp to 167,424 bp. The *A*. *albus* chloroplast (cp) genome contains 113 genes, including 79 protein-coding genes, 30 tRNA genes and 4 rRNA genes. The *A*. *bulbifer* cp genome contains 111 genes, including 78 protein-coding genes, 29 tRNA genes and 4 rRNA genes. *A*. *muelleri* contains 111 and 113 genes, comprising 78 and 80 protein-coding genes, respectively, 29 tRNA genes and 4 rRNA genes. The IR (inverted repeat) region/LSC (long single copy) region and IR/SSC (short single copy) region borders of the four *Amorphophallus* cp genomes were compared. In addition to some genes being deleted, variations in the copy numbers and intron numbers existed in some genes in the four cp genomes. One hundred thirty-four to 164 SSRs (simple sequence repeats) were detected in the four cp genomes. In addition, the highest mononucleotide SSRs were composed of A and T repeat units, and the majority of dinucleotides were composed of AT and TA. SNPs (single nucleotide polymorphisms) and indels (insertion-deletions) were calculated from coding genes and noncoding genes, respectively. These divergences comprising SSRs, SNPs and indel markers will be useful in testing the maternal inheritance of the chloroplast genome, identifying species differentiation and even in breeding programs. Furthermore, the regression of *ndhK* was detected from four *Amorphophallus* cp genomes in our study. Complete cp genome sequences of four *Amorphophallus* species and other plants were used to perform phylogenetic analyses. The results showed that *Amorphophallus* was clustered in *Araceae*, and *Amorphophallus* was divided into two clades; *A*. *albus* and *A*. *konjac* were clustered in one clade, and *A*. *bulbifer* and *A*. *muelleri* were clustered in another clade. Phylogenetic analysis among the *Amorphophallus* genus was conducted based on *matK* and *rbcL*. The phylogenetic trees showed that the relationships among the *Amorphophallus* species were consistent with their geographical locations. The complete chloroplast genome sequence information for the four *Amorphophallus* species will be helpful for elucidating *Amorphophallus* phylogenetic relationships.

## Introduction

The *Amorphophallus* (Araceae) genus contains more than 170 species, mainly distributed throughout Asia and Africa. Twenty-six species were found in Sichuan, Chongqing, Yunan, Guizhou and Hubei Provinces in China^1^. Because the bulbs of *Amorphophallus* are rich in glucomannan, they have been widely used in food, medicine, the chemical industry and so on^2^. In general, the *Amorphophallus* genus produces starch and glucomannan, depending on the species. Much research has focused on *in vitro* propagation systems, due to the accumulation of pathogens from normal asexual reproduction, to increase the yield of *Amorphophallus*^3,4^. The nucleotide sequences (*ITS1*) and plastid sequences (*rbcL* and *matK*) revealed a new subgeneric delineation by large-scale phylogenetic analysis of *Amorphophallus*^5^.

The genome size of *Amorphophallus* is quite large, approximately 20 times larger than the rice genome^6^. Furthermore, large variation exists in the genomic sequences of *Amorphophallus* species. Therefore, sequencing the whole genome of *Amorphophallus* species is very difficult. Complete sequencing of chloroplast (cp) genomes is much easier to achieve in *Amorphophallus* species. The plant chloroplast is a key plastid involved in photosynthesis and carbon fixation^7^. Chloroplast genomes are more conserved than nuclear genomes and contain four important regions: a large single-copy (LSC) region, a small single-copy (SSC) region and a pair of inverted repeats (IRA, IRB)^8^. The cp genome contains important information and genetic markers for phylogenetic and taxonomic analyses between plant species and individuals^9–11^ because of the low rates of polymorphisms, indels and SNPs in cps. More than 800 cp genomes have been sequenced and deposited in the NCBI. The first cp genome was discovered in *Zea mays*^12^, and a complete sequence was determined in *Nicotiana tabacum* and *Marchantia polymorpha*^13,14^. A circular cp genome of *Aquilaria sinensis* was found to be 159,565 bp long and contained 82 protein-coding genes. Zhang *et al*. reported sequences for five *Epimedium* species cp genomes, which provided valuable genetic information for accurately identifying species and assisted in the utilization of *Epimedium* plants^15^. These complete cp genome sequences have been widely used in the development of molecular markers for phylogenetic research^16,17^. Because of the ability for intracellular gene transfer and the conservation, diversity, and genetic basis of chloroplasts, transgene development has allowed for the engineering of high-value agricultural or biomedical products^18^. With the advent of high-throughput sequencing technology, it has become both standard practice and inexpensive to obtain cp genome sequences.

In this study, for the first time, we sequenced the complete cp genomes of four major *Amorphophallus* species using high-throughput sequencing technology and the Illumina HiSeq2500 platform. This study had four aims: (1) determine the size range and structure of four *Amorphophallus* species cp genomes; (2) compare the variations of simple sequence repeats (SSRs) among four major *Amorphophallus* cp genomes; (3) examine the indels and SNPs among four major *Amorphophallus* cp genomes; (4) confirm the phylogenetic relationship among four *Amorphophallus* species, as well as other species, using the complete cp genomes. These results will provide valuable and basic sequence information for taxonomic study and the development of molecular markers for further species identification of *Amorphophallus*. After the completion of the whole cp genome sequence, it is possible to build a database of the species. Based on the differences in the gene sequences of the four cp genomes, a DNA barcode can easily be developed to allow for the building of an efficient platform for postgenomics species research, such as subsequent gene excavation and functional verification of DNA sequence information.

## Results and Discussions

### Organization of four chloroplast genomes

**Figure 1:**
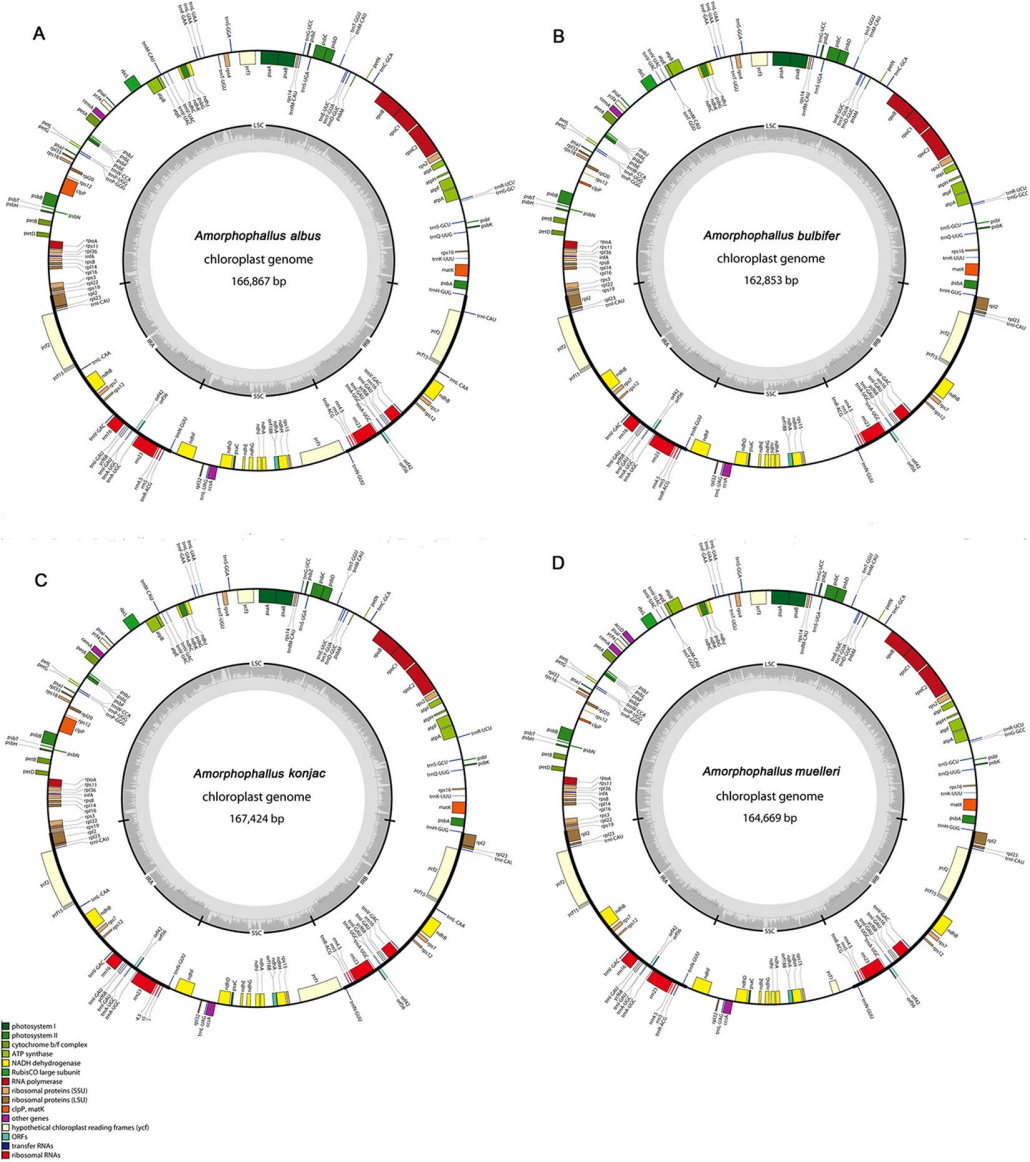
Gene maps of the four *Amorphophallus* cp genomes. (**A**) *A*. *albus*, (**B**) *A*. *bulbifer*, (**C**) *A*. *konjac*, (**D**) *A*. *muelleri*. The annotation of the genome was performed using DOGMA. The genes that are drawn on the outside of the circle are transcribed clockwise, while those inside are transcribed counter clockwise. Genes belonging to different functional groups are color coded. Small single copy (SSC), large single copy (LSC), and inverted repeats (IRa, IRb) are indicated.

Approximately 2G of data for each cp genome was obtained with a 300 bp read length. Gap closing was based on the sequence of the complete cp genome from *Colocasia esculenta* (NC_016753)^19^. The chloroplast genome sequences of the four genomes ranged from 162,853 bp (*A*. *bulbifer*) to 167,424 bp (*A*. *konjac*) (Fig. 1, Table 1). The same typical quadripartite structure was displayed in the four cp genomes. Two IR regions (25,379-26,120 bp) were separated by an LSC region (90,467-92,660 bp) and an SSC region (21,628-22,839 bp) (Table 1). The IRB region was 39 bp longer than the IRA region in the *A*. *konjac* cp genome. The IR/LSC and IR/SSC borders of the four *Amorphophallus* cp genomes were compared (Fig. S1). The variation of the IR/LSC and IR/SSC borders was considered to be the primary mechanism causing the length differences of angiosperm cp genomes^20^. The GC content ranged from 35.39% to 35.90% for the four cp genomes (Table 1). These four *Amorphophallus* cp genome data were deposited in GenBank.

**Table 1:** Summary of the sequencing data for the four Amorphophallus species.

Species	Raw reads no.	Clean reads no.	Gene no.	Protein coding genes no.	tRNA genes no.	rRNA genes no.	Cp genome length (bp)	LSC length (bp)	IRa length (bp)	SSC length (bp)	IRbLength (bp)	GC content (%)
Amorphophallus albus	7383690	6572597	113	79	30	4	166867	92249	25926	22766	25926	35.47
Amorphophallus bulbifer	8590079	7645378	111	78	29	4	162853	90467	25379	21628	25379	35.90
Amorphophallus konjac	7282525	6532968	111	78	29	4	167424	92660	25973	22839	26012	35.39
Amorphophallus muelleri	10581954	8610905	113	80	29	4	164669	90789	26120	21640	26120	35.69

### Divergence hotspots in four chloroplast genomes

The *A*. *albus* cp genome contains 113 genes, including 79 protein-coding genes, 30 tRNA genes and 4 rRNA genes. The *A*. *bulbifer* cp genome contains 111 genes, including 78 protein-coding genes, 29 tRNA genes and 4 rRNA genes. Both the *A*. *konjac* and *A*. *muelleri* cp genomes contain 112 genes, comprising 79 protein-coding genes, 29 tRNA genes and 4 rRNA genes. All of the features are shown in Table 1 and annotated in Fig. 1. All these genes play different roles in the chloroplast, and the classification is shown in Table 2.

**Table 2:** List of genes encoded by the four *Amorphophallus* chloroplast genomes.

Category for genes	Group of genes	Name of genes
Self-replication	rRNA genes	rrn16^a^, rrn23^a^, rrn4.5^a^, rrn5^a^
	tRNA genes	trnA-UGC^*,a^, trnC-GCA, trnD-GUC, trnE-UUC, trnF-GAA, trnfM-CAU, trnG-GCC (Aa, Ab, Am), trnG-UCC, trnH-GUG, trnI-CAU ^a^, trnI-GAU ^*,a^, trnK-UUU, trnL-CAA ^a^ (Aa, Ak), trnL-UAA^*^, trnL-UAG, trnM-CAU ^a^, trnN-GUU ^a^, trnP-GGG, trnP-UGG, trnQ-UUG, trnR-ACG^a^, trnR-UCU, trnS-GCU, trnS-GGA, trnS-UGA, trnT-UGU, trnT-GGU (Aa, Ab ^a^, Ak, Am ^a^), trnV-GAC^,a^, trnV-UAC^*^, trnW-CCA, trnY-GUA
	Small subunit of ribosome	rps2, rps3, rps4, rps7^a^, rps8, rps11, rps12 ^b^, rps14, rps15, rps16, rps18, rps19
	Large subunit of ribosome	rpl2 (Aa^*^, Ab^*, a^, Am^*, a^, Ak^*, a^), rpl20, rpl23 (Aa^*^, Ab^a^, Am^*, a^, Ak^*, a^), rpl33, rpl36, rpl14, rpl16, rpl22, rpl32
	RNA polymerase	rpoA, rpoB, rpoC1^*^, rpoC2
Phytosynthesis	NADH-dehydrogenase	ndhA ^a^, ndhB^*, a^, ndhC, ndhD, ndhE, ndhF, ndhG, ndhH, ndhI, ndhK, ndhJ psbG,
	Photosystem I	psaA, psaB, psaC, psaI, psaJ, ycf3^**^
	Photosystem II	psbA, psbB, psbC psbD, psbE (Aa, Ab, Am), psbF (Aa, Ab, Am, Ak^*^), psbH, psbI, psbJ, psbK, psbL, psbM, psbN, psbT, psbZ
	Cytochrome b/f complex	petA, petB, petD, petL, petG, petN,
	ATP synthase	atpA, atpB, atpE, atpF^*^, atpH, atpI
	Large subunit to rubisco	rbcL
Other genes	Maturase	matK
	Protease	clpP(Aa^**^, Ab, Am, Ak^**^)
	Envelope membrane protein	cemA
	Subunit of Acetyl-CoA-Carboxylase	*accD*(*Am*)
	c-type cytochrome synthesis gene	ccsA
	Translational initiation factor	infA^**^
Genes of unknown function	Open Reading Frames	ycf1 (Aa, Ak, Am), ycf2 (Aa^a^, Ab^a^, Am^*, a^, Ak^a^), ycf4,
Putative pseudogenes		ycf15 (Aa^*, a^, Ab^a^, Am^*, a^, Ak^a^), ycf68 ^a^

^a^Gene with two copies; ^b^Gene with three copies; ^*^Gene with one intron; ^**^Gene with two introns.

( )Gene existed in which species cp genome as well as copy number and intron number in each cp genome.

Aa, Amorphophallus albus cp genome; Ab, Amorphophallus bulbifer cp genome; Ak, Amorphophallus konjac cp genome; Am, Amorphophallus muelleri cp genome.

The estimated deletion of some genes was detected in some *Amorphophallus* cp genomes (Table 3). The *ycf1* gene was present in three cp genomes but not in the *A*. *bulbifer* cp genome. Another gene named *trnL-CAA* appeared in the *A*. *albus* and *A*. *konjac* cp genomes. The *trnG-GCC* gene was lost in the *A*. *konjac* cp genome. The *accD* gene was found only in the *A*. *muelleri* cp genome, and *psbE* was missing only in the *A*. *konjac* cp genome. The *rpl2* and *rpl23* genes were annotated in the IRA and IRB regions of the four cp genomes, but they were only found in the IRA region and were lost in the IRB region in the *A*. *albus* cp genome.

**Table 3:** Summary of genes estimate deletion in the four *Amorphophallus* cp genomes.

Gene	Species (cp genome)				Region
	*A*. *albus*	*A*. *bulbifer*	*A*. *konjac*	*A*.*muelleri*	
*ycf1*	+	−	+	+	SSC
*rpl23*	IRA+; IRB−	+	+	+	IR
*rpl2*	IRA+; IRB−	+	+	+	IR
*trnL-CAA*	+	−	+	−	IR
*trnG-GCC*	+	+	−	+	LSC
*accD*	−	−	−	+	LSC
*psbE*	+	+	−	+	LSC

+ Gene existing. − Gene estimate deletion.

In addition to some genes being deleted, variations in the copy numbers and intron numbers of some genes were also found in the four cp genomes. Eight protein-coding genes, four rRNA genes, nine tRNA genes and two putative genes were present in two copies. Moreover, *trnT-GGU* was found to have two copies only in the *A*. *bulbifer* and *A*. *muelleri* cp genomes. In addition, three copies of the *rps12* gene were found. Fifteen genes contained introns, including four tRNA genes, ten protein-coding genes and one putative gene. The *psbF* and *ycf2* genes containing one intron were only found in the *A*. *muelleri* cp genome. There were no introns in the *clpP* gene in the *A*. *bulbifer* and *A*. *muelleri* cp genomes, but there were two introns in this gene in the *A*. *albus* and *A*. *konjac* cp genomes, while *ycf3* and *infA* had two introns in each of the four cp genomes. All of the above divergences are shown in Table 2. The development of molecular markers for the identification of *Amorphophallus* species was much easier based on the divergence hotspot regions of the four *Amorphophallus* cp genomes.

### COG analysis

COG (clusters of orthologous groups of proteins) and KOG (eukaryotic ortholog groups) are based on the relationship between orthologous genes in the NCBI annotation system for prokaryotes and eukaryotes^21^, respectively. Homologous genes from different species can be divided into different ortholog clusters combining evolutionary relationships. There are 4,873 categories in COG and 4,852 in KOG. Genes that are orthologs have the same function, and the functional annotation can be inferred to other members of the same COG/KOG clusters. All of the genes from the four cp genomes were classified into six categories: energy production and conversion; translation, ribosomal structure and biogenesis; posttranslational modification, protein turnover and chaperones; transcription; carbohydrate transport and metabolism; and lipid metabolism. The number of genes classified under each function in the four *Amorphophallus* genomes is shown in Fig. S2.

### SSR polymorphisms and SNP/Indel analysis

SSRs are important molecular markers for plant evolutionary and ecological studies^15^, and they are widely present in the cp genome. With MISA analysis, 134–164 SSRs were detected in the four cp genomes (Table 4**)**. Among these SSRs, mono-, di-, tri-, tetra-, and hexanucleotides were detected. The mononucleotide SSRs were most common, with 70.15% of the SSRs observed in *A*. *bulbifer*. In addition, most of the mononucleotide SSRs were composed of A and T repeat units, and the majority of the dinucleotides were composed of AT and TA. The cp SSRs are normally composed of short polyA or polyT repeats^22^. Higher contents of A/T and AT/TA repeats in cp SSRs were also detected in the *Metasequoia glyptostroboides* cp genome^23^. Hexanucleotide repeat unit SSRs were in the *A*. *muelleri* cp genome only at a portion of 2.08%. In short, the cp SSRs represented rich variation and were absolutely useful for polymorphism analysis in the *Amorphophallus* species.

**Table 4:** Simple sequence repeats (SSRs) in the four Amorphophallus species cp genomes.

Species	SSR loci no.	P1 loci no.	P2 loci no.	P3 loci no.	P4 loci no.	P5 loci no.	P6 loci no.
Amorphophallus albus	163	86	49	27	1	/	/
Amorphophallus bulbifer	134	94	25	14	1	/	/
Amorphophallus konjac	164	82	51	30	1	/	/
Amorphophallus muelleri	144	80	45	15	1	/	3

Using the *A*. *albus* cp genome as the reference sequence, we compared the SNP/indel loci of the four cp genomes. SNP markers were detected in 65 protein-coding genes in *A*. *bulbifer*, *A*. *konjac* and *A*. *muelleri* cp genomes. Eleven genes were in the SSC region, and 54 genes were in the LSC region, indicating that the protein-coding genes in the IR region were more conserved. These 65 genes were divided into four categories according to their different functions in plant chloroplasts, including photosynthetic apparatus, photosynthetic metabolism, gene expression, and other genes. Nine hundred sixty-nine and 943 SNP markers were detected between *A*. *albus* and *A*. *bulbifer* in protein-coding genes and noncoding regions, respectively. One hundred and four and 176 SNP markers were detected between *A*. *albus* and *A*. *konjac* in protein-coding genes and noncoding regions, respectively. Nine hundred and seventy-eight and 926 SNP markers were detected between *A*. *albus* and *A*. *muelleri* in protein-coding genes and noncoding regions, respectively. The SNPs in the *A*. *konjac* cp genome were significantly fewer than those in the *A*. *bulbifer* and *A*. *muelleri* cp genomes. One hundred and fifty-nine SNP sites were found in *Oryza*. *sativa* and *Oryza*. *nivara* chloroplast genomes^24^, 591 SNP markers were detected between the *Solanum tuberosum* and *S*. *bulbocastanum* plastomes^25^, and 464 were detected between the plastomes of *P*. *ginseng* and *P*. *notoginseng*^26^.

**Figure 2:**
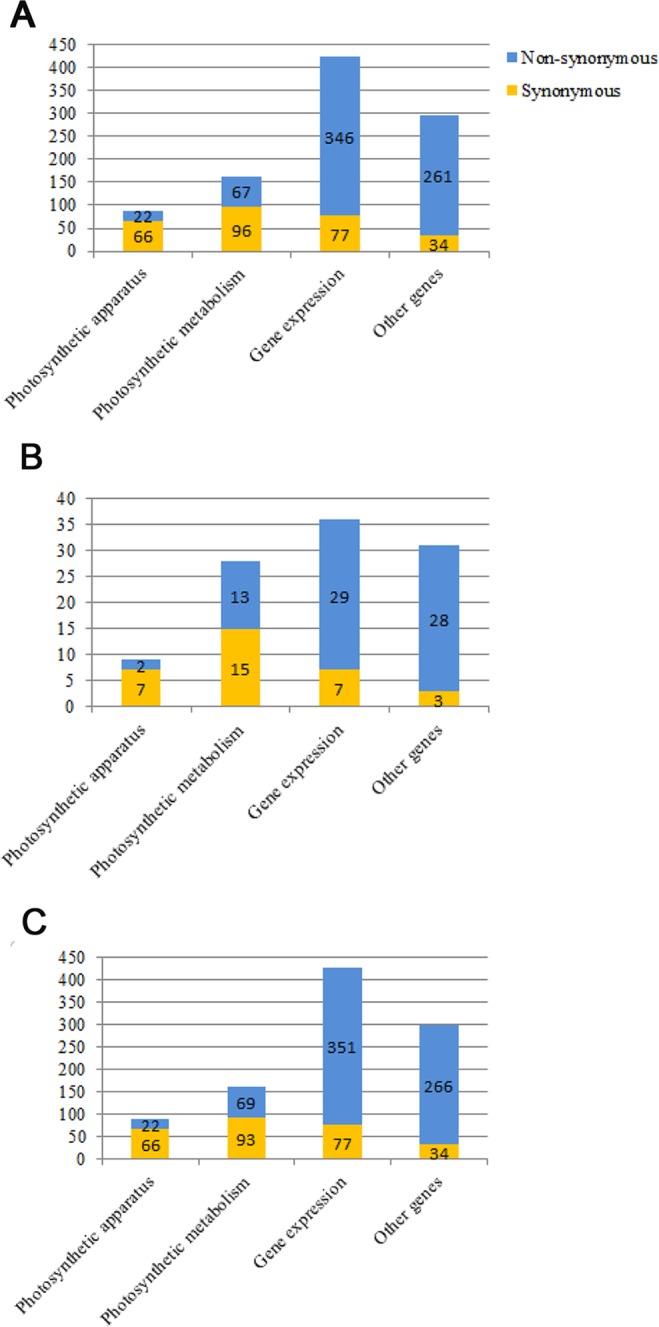
SNPs statistics of *A*. *bulbifer*, *A*. *konjac* and *A*. *muelleri* cp genomes. The *Amorphophallus albus* cp genome was used as the reference sequence for SNPs analyses for the other three cp genomes. SNPs belonging to different type groups are color coded. (**A**) Number of SNPs in the *A*. *bulbifer* cp genome sequence. (**B**) The number of SNPs in the *A*. *konjac* cp genome sequence. (**C**) The number of SNPs in the *A*. *muelleri* cp genome sequence.

All of the SNPs were classified into two types, including synonymous (S) and nonsynonymous (N) (Table 5, Fig. 2). For the 969 and 978 SNP markers in the gene coding regions of the *A*. *bulbifer* and *A*. *muelleri* cp genomes, respectively, 696 and 708 belonged to the nonsynonymous type, and 273 and 270 belonged to the synonymous type. Synonymous and nonsynonymous SNP makers from the gene coding genes shared very similar numbers in these two cp genomes. There were 32 synonymous SNPs and 72 nonsynonymous SNPs in the protein coding regions of the *A*. *konjac* cp genome. Forty-eight nonsynonymous and 47 synonymous SNP sites were detected in the *Machilus* cp genome, implying that a substitution constraint mechanism existed^27^. Genes *ycf3*, *rpoC1* and *clpP* were detected with SNP markers in their introns. Six, 1 and 6 SNP markers were found in one intron from *rpoC1*; 6, 1 and 5 SNP markers were found in one intron from *ycf3*; 23, 7 and 25 SNP markers were found in two introns from *clpP* in the *A*. *bulbifer*, *A*. *konjac and A*. *muelleri* cp genomes, respectively. *ClpP* and *ycf1* were the variation hotspots for SNPs and indels, and they were usually used for investigating sequence variation in seed plants^28,29^.

**Table 5:** Comparisons of mutation changes, number of synonymous (S) and nonsynonymous (N) substitutions per gene of protein coding cp genes among *A*. *bulbifer*, *A*. *konjac* and *A*. *muelleri*.

	Gene	*A. bulbifer*		*A. konjac*		*A. muelleri*		Region
		S	N	S	N	S	N	
Photosynthetic apparatus	*psbA*	9	0	0	0	9	0	LSC
	*psbB*	7	2	0	0	8	2	LSC
	*psbD*	2	1	0	0	2	1	LSC
	*psbC*	3	1	0	0	3	1	LSC
	*psbF*	2	0	1	0	2	0	LSC
	*psbG*	2	5	1	0	2	5	LSC
	*psbH*	2	0	0	0	2	0	LSC
	*psbI*	3	1	0	0	3	1	LSC
	*psbL*	1	0	1	0	1	0	LSC
	*psbN*	1	0	0	0	1	0	LSC
	*psaA*	10	4	0	0	10	4	LSC
	*psaB*	10	2	2	2	9	2	LSC
	*psaC*	1	0	0	0	2	0	SSC
	*psaI*	1	0	0	0	1	0	LSC
	*psaJ*	0	1	0	0	0	0	LSC
	*petA*	3	3	1	0	3	3	LSC
	*petB*	3	0	0	0	3	0	LSC
	*petD*	2	1	0	0	3	1	LSC
	*petG*	1	0	1	0	1	0	LSC
	*petL*	0	0	0	0	0	1	LSC
	ycf3*	3	1	0	0	1	1	LSC
	**Total**	**66**	**22**	**7**	**2**	**66**	**22**	
Photosynthetic metabolism	*atpA*	9	5	0	1	8	6	LSC
	*atpB*	7	5	0	0	8	5	LSC
	*atpE*	2	1	0	0	2	1	LSC
	*atpF*	0	4	0	0	0	4	LSC
	*atpH*	1	1	0	0	1	1	LSC
	*atpI*	3	3	2	1	3	3	LSC
	*ndhA*	10	12	2	4	10	12	SSC
	*ndhC*	1	2	1	1	1	2	LSC
	*ndhD*	17	6	2	0	16	5	SSC
	*ndhE*	4	0	0	0	4	0	SSC
	*ndhF*	13	14	2	4	11	16	SSC
	*ndhG*	2	4	1	2	2	5	SSC
	*ndhH*	11	7	3	0	12	6	SSC
	*ndhI*	3	1	0	0	3	1	SSC
	*ndhJ*	1	0	0	0	0	0	LSC
	*rbcL*	12	2	2	0	12	2	LSC
	**Total**	**96**	**67**	**15**	**13**	**93**	**69**	
Gene expression	*rpoA*	3	5	0	0	2	5	LSC
	*rpoB*	10	23	1	3	11	22	LSC
	*rpoC2*	18	41	3	8	17	41	LSC
	rpoC1*	12	13	1	2	14	13	LSC
	*rps2*	4	30	1	1	4	31	LSC
	*rps3*	1	40	0	1	1	39	LSC
	*rps4*	3	22	0	1	3	24	LSC
	*rps8*	1	5	0	0	1	5	LSC
	*rps11*	7	30	0	0	7	33	LSC
	*rps12*	2	5	0	0	1	5	LSC
	*rps14*	2	11	0	1	2	12	LSC
	*rps15*	0	13	0	5	0	13	SSC
	*rps16*	0	6	0	0	0	8	LSC
	*rps18*	1	14	0	3	1	14	LSC
	*rps19*	2	27	1	1	3	26	LSC
	*rpl14*	1	19	0	0	0	17	LSC
	*rpl16*	1	10	0	0	1	10	LSC
	*rpl20*	2	11	0	1	2	10	LSC
	*rpl22*	4	8	0	1	4	8	LSC
	*rpl32*	1	2	0	0	1	2	SSC
	*rpl33*	1	2	0	1	1	3	LSC
	*rpl36*	1	9	0	0	1	10	LSC
	**Total**	**77**	**346**	**7**	**29**	**77**	**351**	
Other genes	*ycf1*	12	170	0	24	12	174	SSC
	*ycf4*	2	3	0	0	2	3	LSC
	*cemA*	2	6	0	1	2	6	LSC
	clpP*	15	73	2	3	14	74	LSC
	*infA*	1	2	0	0	1	2	LSC
	*cemA*	2	7	1	0	3	7	LSC
	**Total**	**34**	**261**	**3**	**28**	**34**	**266**	

^*^SNP markers were detected in their introns.

cpSSR and SNP markers will be useful in testing maternal inheritance of the cp genome, identifying species differentiation and even in breeding programs^30^. cpSSRs have been demonstrated to be useful in gene flow studies to estimate seed and pollen contribution^31^ and in phylogeographic analyses^32^.

**Figure 3:**
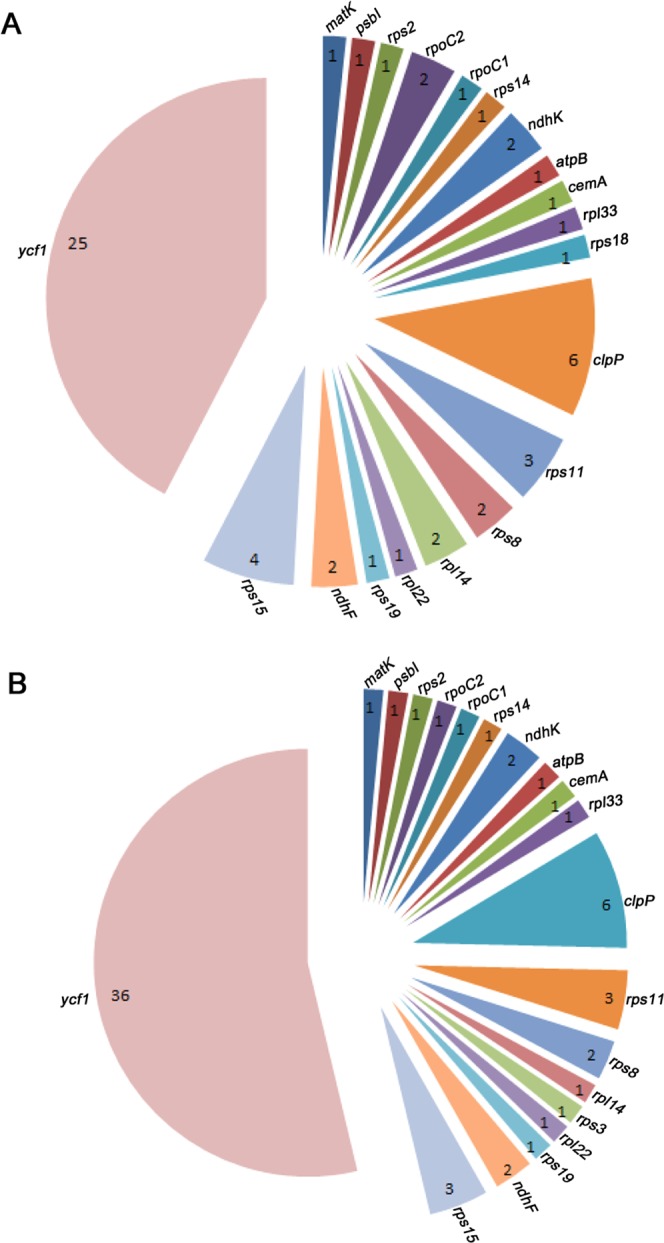
InDels statistics of *A*. *bulbifer* and *A*. *muelleri* cp genomes. The *Amorphophallus albus* cp genome was used as the reference sequence for InDels analyses for the other three cp genomes. InDels belonging to different coding genes are color coded. Only three InDels were detected in the *A*. *konjac* cp genome, so the statistics results are shown in the main text. (**A**) The number of InDels of each coding gene in the *A*. *bulbifer* cp genome sequence, (**B**) The number of InDels of each coding gene in the *A*. *muelleri* cp genome sequence.

Twenty-two protein-coding genes from three *Amorphophallus* cp genomes contained indels (Table 6). Only two coding genes were detected to contain indels in the *A*. *konjac* cp genome; one indel was in *rps15*, and two indels existed in *ycf1*. The indel numbers of each coding gene from the *A*. *bulbifer* and *A*. *muelleri* cp genomes are shown in Fig. 3A,B. The gene *ycf1* was a hotspot for indel variation, and almost half of the number of indels existed in this gene (Fig. 3). Such mutational loci in cp genomes showed highly variable regions in the genomes.

**Table 6:** Comparisons of InDels of protein coding cp genes among *A*. *bulbifer*, *A*. *konjac* and *A*. *muelleri*.

Gene	motif	size	Direction
*matK*	Ab: accaaataccaa	12	Deletion
	Am: accaaataccaa	12	Deletion
*psbI*	Ab: aaaa	4	Insertion
	Am: aataa	5	Insertion
*rps2*	Ab: cttttt	6	Insertion
	Am: tctttt	6	Insertion
*rpoC2*	Ab: aat	3	Deletion
*rpoC2*	Ab: act	3	Insertion
	Am: cat	3	Insertion
*rpoC1*	Ab: a	1	Insertion
	Am: tta	3	Insertion
*rps14*	Ab: tttttt	6	Insertion
	Am: tttttt	6	Insertion
*psbG*	Ab: ttt	3	Deletion
	Am: ttt	3	Deletion
*ndhK*	Ab: aaaaaaaa	8	Insertion
	Am: ttggaattgggagaataaccca	22	Insertion
*atpB*	Ab: a	1	Insertion
	Am: a	1	Insertion
*cemA*	Ab: tgat	4	Deletion
	Am: tgat	4	Deletion
*rpl33*	Ab: a	1	Insertion
	Am: t	1	Deletion
*rps18*	Ab: aaaaaaaaaa	10	Insertion
*clpP*	Ab: t	1	Insertion
	Am: a	1	Deletion
*clpP*	Ab: t	1	Insertion
	Am: t	1	Insertion
*clpP*	Ab: c	1	Deletion
	Am: c	1	Deletion
*clpP*	Ab: c	1	Deletion
	Am: c	1	Deletion
*clpP*	Ab: g	1	Insertion
	Am: g	1	Insertion
*clpP*	Ab: g	1	Deletion
	Am: g	1	Deletion
*rps11*	Ab: a	1	Insertion
	Am: a	1	Insertion
*rps11*	Ab: t	1	Deletion
	Am: t	1	Deletion
*rps11*	Ab: c	1	Insertion
	Am: c	1	Insertion
*rps8*	Ab: tttccctag	9	Deletion
	Am: tttccctag	9	Deletion
*rps8*	Ab: cggg	4	Insertion
	Am: ctggttg	7	Insertion
*rpl14*	Ab: aaaacg	6	Insertion
	Am: aatatacg	8	Insertion
*rpl14*	Ab: aatttt	6	Insertion
*rps3*	Am: ta	2	Insertion
*rpl22*	Ab: c	1	Insertion
*rpl22*	Am: c	1	Insertion
*rps19*	Ab: aaa	3	Deletion
	Am: aaa	3	Deletion
*ndhF*	Ab: tttgaca	7	Deletion
*ndhF*	Ab: aaa	3	Insertion
	Am: aatcca	6	Insertion
*ndhF*	Am: ttttttt	7	Insertion
*rps15*	Ab: g	1	Deletion
*rps15*	Ab: gttgaattaacg	12	Deletion
*rps15*	Am: cgaga	5	Deletion
*rps15*	Ab: gtttttg	7	Deletion
*rps15*	Am: aaa	3	Deletion
*rps15*	Am: g	1	Deletion
*rps15*	Ab: aaaa	4	Insertion
*rps15*	Ak: aaaa	4	Insertion
*ycf1*	Ab: agg	3	Insertion
	Am: tagg	4	Insertion
*ycf1*	Ab: ctttt	5	Insertion
	Am: tcttt	5	Insertion
*ycf1*	Am: gttttttaa	9	Deletion
*ycf1*	Am: ttatta	6	Insertion
*ycf1*	Ab: aaaaaaaccccccccggggttttttttttt	30	Insertion
	Am: tcgtccaggcatcaatatcgctatttattt	30	Insertion
*ycf1*	Ab: aacctt	6	Insertion
	Am: ctcaat	6	Insertion
*ycf1*	Ab: aaaaccgg	8	Insertion
	Am: atatcgatatcg	12	Insertion
*ycf1*	Ab: ccatataga	9	Deletion
	Am: ccatataga	9	Deletion
*ycf1*	Ab: ttttctgtg	9	Deletion
	Am: ttttctgtg	9	Deletion
*ycf1*	Ab: aagttt	6	Insertion
	Am: gtatat	6	Insertion
*ycf1*	Ab: cat	3	Deletion
	Am: cat	3	Deletion
*ycf1*	Ab: ccgtaataa	9	Deletion
	Am: ccgtaataa	9	Deletion
*ycf1*	Ab: acc	3	Insertion
	Am: tctatc	6	Insertion
*ycf1*	Ab: aaaacgttttttttt	15	Insertion
*ycf1*	Am: tga	3	Insertion
*ycf1*	Am: ttc	3	Insertion
*ycf1*	Am: tt	2	Insertion
*ycf1*	Am: t	1	Insertion
*ycf1*	Ab: act	3	Insertion
	Am: cat	3	Insertion
*ycf1*	Ab: c	1	Insertion
	Am: ttc	3	Insertion
*ycf1*	Ab: a	1	Insertion
	Am: tta	3	Insertion
*ycf1*	Am: g	1	Deletion
*ycf1*	Ab: cctttt	6	Insertion
	Am: tccttt	6	Insertion
*ycf1*	Ab: gattttcgccatcgtacttt	20	Deletion
	Am: gattttcgccatcgtacttt	20	Deletion
*ycf1*	Ak: atcgatctttagattttcgcc	21	Deletion
*ycf1*	Ab: g	1	Deletion
	Am: g	1	Deletion
*ycf1*	Am: tctttctttctctttttctttct	23	Insertion
*ycf1*	Am: tt	2	Insertion
*ycf1*	Am: tc	2	Insertion
*ycf1*	Ab: cccccgtttttttttttttttttt	24	Insertion
*ycf1*	Ak: tcagaa	6	Insertion
*ycf1*	Ab: ctt	3	Insertion
*ycf1*	Am: gttcattattatcattatcattat	24	Insertion
*ycf1*	Am: a	1	Insertion
*ycf1*	Am: atc	3	Insertion
*ycf1*	Am: attatcattatcatt	15	Insertion
*ycf1*	Ab: g	1	Insertion
	Am: g	1	Insertion
*ycf1*	Ab: a	1	Insertion
	Am: a	1	Insertion
*ycf1*	Ab: c	1	Insertion
	Am: c	1	Insertion
*ycf1*	Ab: c	1	Insertion
	Am: ttc	3	Insertion
*ycf1*	Ab: gg	2	Insertion
	Am: gg	2	Insertion
*ycf1*	Ab: c	1	Insertion
	Am: c	1	Insertion
*ycf1*	Am: atcattcctgat	12	Insertion
*ycf1*	Am: cg	2	Insertion
*ycf1*	Am: ga	2	Insertion
*ycf1*	Am: tc	2	Insertion
*ycf1*	Ab: ttaaaaagg	9	Deletion
	Am: ttaaaaagg	9	Deletion
*ycf1*	Ab: aaccccgggg	10	Insertion
	Am: ttgttccagttgttccgg	18	Insertion
*ycf1*	Am: caa	3	Insertion

Ab Amorphophallus bulbifer cp genome, Ak Amorphophallus konjac cp genome, Am Amorphophallus muelleri cp genome

### *ndhK* regression in four *Amorphophallus* species cp genomes

The *ndhK* gene was a new gene represented in the four *Amorphophallus* species cp genomes. It was 744 bp in length in the *A*. *albus* and *A*. *konjac* cp genomes, and 741 bp in length in the *A*. *bulbifer* and *A*. *muelleri* cp genomes. The gene *ndhK* is present in a novel protein complex of the thylakoid membrane and shows homology to a mitochondrial gene that encodes a subunit of the NADH-ubiquinone oxidoreductase of the mitochondria^33^. *ndhK* was reported as a gene encoding a subunit of PSII, but later, this protein was classified as a subunit of NADH dehydrogenase, and the gene has been renamed *ndhK*^34,35^. In many plants, such as *Glycine max*, *Epimedium acuminatum*, *Psilotum nudum*, *Machilus yunnanensis*, *Actinidia chinensis*, *Veronica persica*, and *Aquilaria sinensis* (Lour.) Gilg., *ndhK* was lost from their cp genomes^15,18,27,36–39^. *ndhK* has been found in the *Paramecium aurelia* mitochondrial (mt) genome^40^. The presence of this gene in the mt genome raises interesting questions concerning its evolutionary origin. The gene *ndhK* may play a crucial role in photosynthesis in four *Amorphophallus* species, and its presence in the cp genomes can be used as a marker for distinguishing them from other family species.

### Phylogenetic analysis

**Figure 4:**
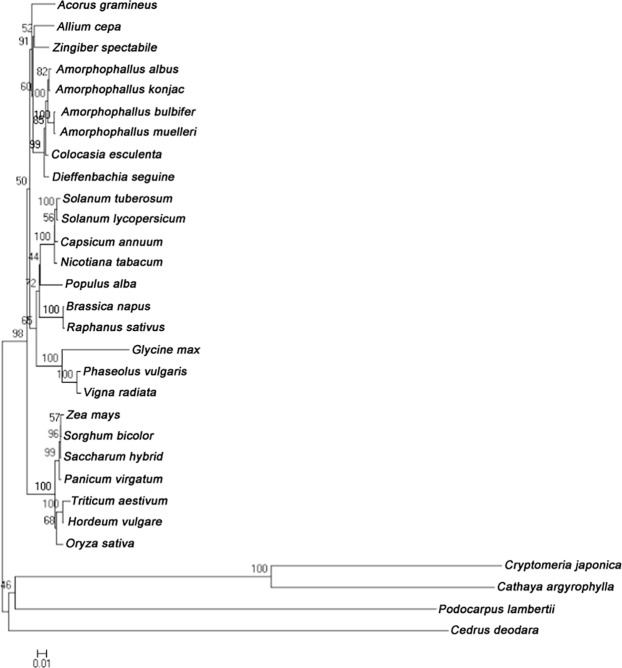
Phylogenetic tree based on 30 complete cp genome sequences.

Phylogenetic analysis of *Amorphophallus* species has been reported using different aspects, such as several chloroplast genes^41^, two chloroplast genes, *leafy* intron sequences^42^, plastid DNA markers and fingerprinting^43^. These studies simply demonstrated *Amorphophallus* sample species relationships and did not include the four *Amorphophallus* species that are the major commercial cultivation species used in our study. In addition, whole chloroplast sequences were much more accurate than individual gene sequences for phylogenetic analysis. In the present study, complete cp genomes sequences of four *Amorphophallus* species and other plants (Table S1) were used to perform phylogenetic analyses (Fig. 4). The clade of the four species of *Amorphophallus* was grouped with other *Araceae* species as expected. *A*. *albus* and *A*. *konjac* were clustered into one clade, and *A*. *bulbifer* and *A*. *muelleri* were clustered into another clade. These results showed that *A*. *albus* and *A*. *konjac* had a close relationship, and *A*. *bulbifer* and *A*. *muelleri* were closely related. The *matK* and *rbcL* genes were also used for phylogenetic analysis among the *Amorphophallus* genus (Figs S3 and S4**)**. Both of the phylogenetic trees indicated that the *Amorphophallus* species were grouped into three major clades named Africa, southeast Asia, and Continental Asia. The Continental Asia clade covered the taxa distributed from India to China and Thailand, which were subdivided into two subclades, Continental Asia I and II. The four *Amorphophallus* species in our study were all derived from the Chinese mainland; *A*. *albus* and *A*. *konjac* were grouped as Continental Asia I, and *A*. *bulbifer* and *A*. *muelleri* were grouped as Continental Asia II. The first two species came from the central region of China, and the other two species were collected from the southern region of China near Burma. The *matK* and *rbcL* genes well supported clades in consensus trees and the resolution of ingroup relationships within *Amorphophallus*^44^. All the results suggested that the relationship in *Amorphophallus* was consistent with the biogeographical distribution. *A*. *konjac* and *A*. *bulbifer* were also classified in two different clades by Sedayu^42^. *A*. *albus* and *A*. *konjac* have the same chromosome number (2N = 2X = 26), while *A*. *bulbifer and A*. *muelleri* are triploid (3N = 3X = 39). The propagation coefficient of *A*. *albus* and *A*. *konjac* did not exceed single digits, while the propagation coefficient in *A*. *bulbifer* and *A*. *muelleri* increased significantly because of aerial bulbs growing in the stems. The aerial bulbs diminish the need for sexual reproduction and lead to a significantly increased reproductive capacity. In many cases, the evolutionary process is closely linked with the reproduction system of the species. *A*. *muelleri* and *A*. *bulbifer* reproduce, thus far, through apomictic processes. The corm of *A*. *bulbifer* is light red, and that of *A*. *muelleri* is light yellow. These phenotypes also demonstrated the relationship among the four *Amorphophallus* species. The sequenced cp genomes of the four *Amorphophallus* species provide a large amount of genetic information for phylogenetic analysis and taxonomic study.

## Conclusion

We sequenced the chloroplast genomes of four *Amorphophallus* plants: *A*. *albus*, *A*. *bulbifer*, *A*. *konjac*, and *A*. *muelleri*. We annotated the four cp genomes and analyzed the structural divergence among the four cp genomes; moreover, we identified the SSR loci and SNPs in protein-coding genes. These SSRs and SNPs could be selected for use in developing markers and in phylogenetic analysis. Comparing the cp genomes among some plants suggested that *psbG* regressed in the *A*. *albus*, *A*. *konjac*, *A*. *bulbifer* and *A*. *muelleri* cp genomes. We also detected that some genes and introns were lost, in addition to copy differences of some genes among the four cp genomes. The results of SNP detection demonstrated that very few of the SNPs were identified between the *A*. *albus* and *A*. *konjac* cp genomes; on the contrary, a large number of SNPs between *A*. *bulbifer* and *A*. *muelleri* were identified when the *A*. *albus* cp genome was used as the reference sequence. Interestingly, the SNPs were almost the same in the *A*. *bulbifer* and *A*. *muelleri* cp genomes. The indel results were very similar between *A*. *albus* and *A*. *konjac* because only three indels were detected in the *A*. *konjac* cp genome. In addition, phylogenetic analysis using complete cp genome sequences showed that *A*. *albus* and *A*. *konjac* were in a clade and *A*. *bulbifer* and *A*. *muelleri* were in a different clade. The clustering analysis results verified the results of the SNP data. All the data will be very helpful in further research on *Amorphophallus* plants and chloroplasts and in expanding our understanding of the evolutionary history of the *Amorphophallus* cp genomes. All of these divergences in the four cp genomes were significant for taxonomic and evolutionary studies, as well as for genetic engineering developments in the future.

## Methods

### Plant material preparation and sequencing

Fresh young leaves of *A*. *albus*, *A*. *bulbifer*, *A*. *konjac* and *A*. *muelleri* were collected from live individuals at the greenhouse of Wuhan University in China. Five micrograms of cp DNA was isolated from leaves and sheared into 300 bp DNA fragments using a Covaris M220 (Covaris, United States). NEB Next ® UltraTM DNA Library Prep Kit for Illumina (NEB, United States) was used to build the library after DNA fragmentation. The genomic DNA of four species was sequenced on a single HiSeq2500 flow cell lane (Illumina Inc.) by the Chinese National Human Genome Center (http://www.chgc.sh.cn/), Shanghai, China.

### Plant cp genome assembly and annotation

Trimmomatic v 0.32^45^ was used for raw data processing, and the resulting clean data were used for assembly and post analysis. Fastqc v0.10.0^46^ was used to evaluate the quality of the data visually. Velvet v1.2.07^47^ was used to assemble the clean data, and the complete chloroplast genome sequence was obtained after gap closing. DOGMA^48^ was used to annotate the cp genomes and predict the rRNA/tRNA of *A*. *albus*, *A bulbifer*, *A*. *konjac*, and *A*. *muelleri*. COGs (clusters of orthologous groups of proteins) were analyzed through rpsblast v2.2.30+^49^. The circular cp genome maps were drawn using the OrganellarGenomeDRAW program^50^.

### Mutation events analysis

To compare the mutations among the four complete cp genomes, MISA and MUMMER 3.23 software was used for SSR and SNP/indel analyses, respectively. The *A*. *albus* cp genome was used as a reference sequence for SNP/indel analyses. Definition of microsatellites (unit size/minimum number of repeats): (1/10) (2/5) (3/4) (4/4) (5/4) (6/4).

### Phylogenetic analysis

We selected twenty-six cp genomes (Table S1), representing the nine families, for phylogenetic analysis. The *matK* and *rbcL* genes were used for phylogenetic analysis among the *Amorphophallus* genus, and the selected species are shown in Tables S2 and S3. MEGA 6.06 software was used for building the evolutionary tree. The analysis was carried out based on the complete cp DNA sequences.

## Supplementary information

Figure S1-S4, Table S1-S3

## Data Availability

All data generated or analyzed during this study are included in this published article (and its Supplementary Information files).
