# Supplementary material for: Comparative analysis of complete chloroplast genome sequences of four major Amorphophallus species
Source: Sci Rep. 2019 Jan 28;9:809. doi: 10.1038/s41598-018-37456-z (PMC6349887; doi:10.1038/s41598-018-37456-z)
Supplement: Supplementary file 1 — Figure S1-S4, Table S1-S3 [file 41598_2018_37456_MOESM1_ESM.pdf]

Comparative analysis of complete chloroplast genome sequences of four major Amorphophallus species

Erxi Liu[1,2], Chaozhu Yang[2], Jiangdong Liu[1], Surong Jin[3], Nunung Harijati[4], Zhongli Hu[1], Ying Diao[1] and Lingling Zhao*[1]

[1] State Key Laboratory of Hybrid Rice, Lotus Engineering Research Center of Hubei Province, College of Life Science, Wuhan University, Wuhan, Hubei 430072, P. R. China, [2] Institute of Konjac, Enshi Academy of Agricultural Sciences, Enshi, P. R. China, [3] School of Chemistry ,Chemical Engineering and Life Science,Wuhan University of Technology, Wuhan, 430070, P. R. China, [4] Department of Biology, Faculty of Mathematics and Natural Sciences, Brawijaya University, Jl. Veteran Malang, 65145, Indonesia.

**Correspondence to* zhaolingling@whu.edu.cn

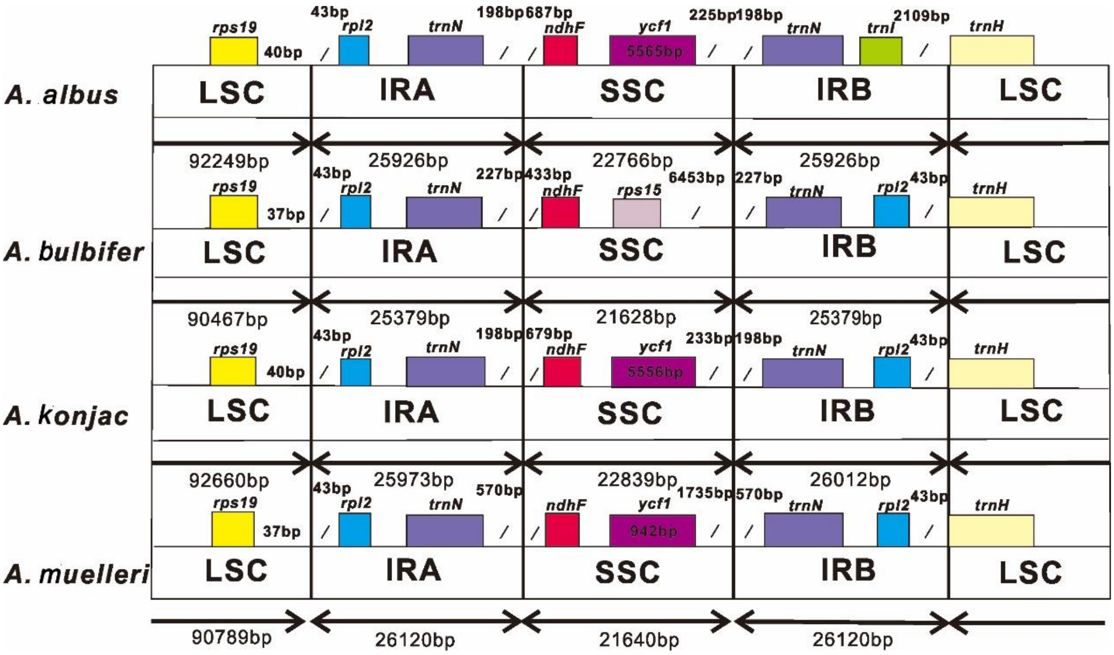

Figure S1 Comparisons of LSC, SSC and IR region borders among the four Amorphophallu cp genomes.

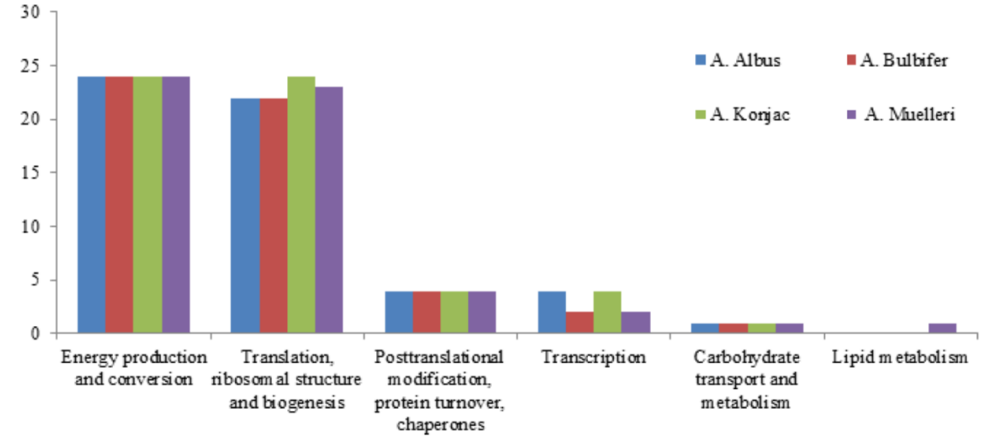

Figure S2 COG function classification of genes from four Amorphophallu cp genomes. A. albus, A. bulbifer, A. konjac and A. muelleri are color coded.

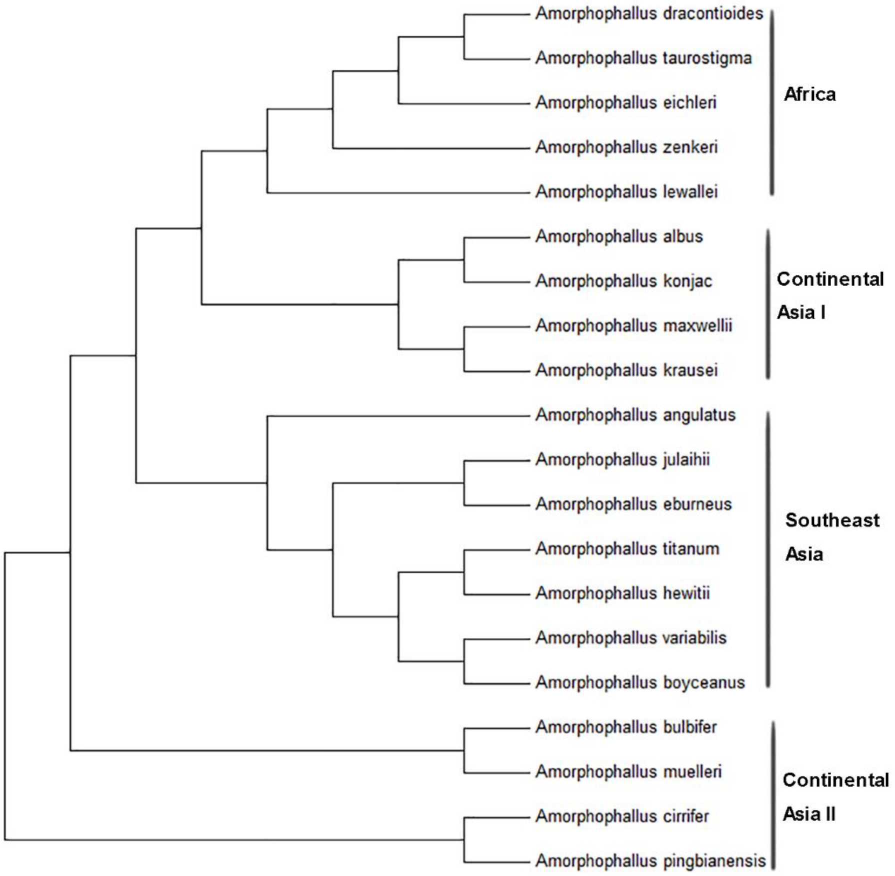

Figure S3 Phylogenetic tree based on matK gene sequences from our four Amorphophallus species and other Amorphophallus genus.

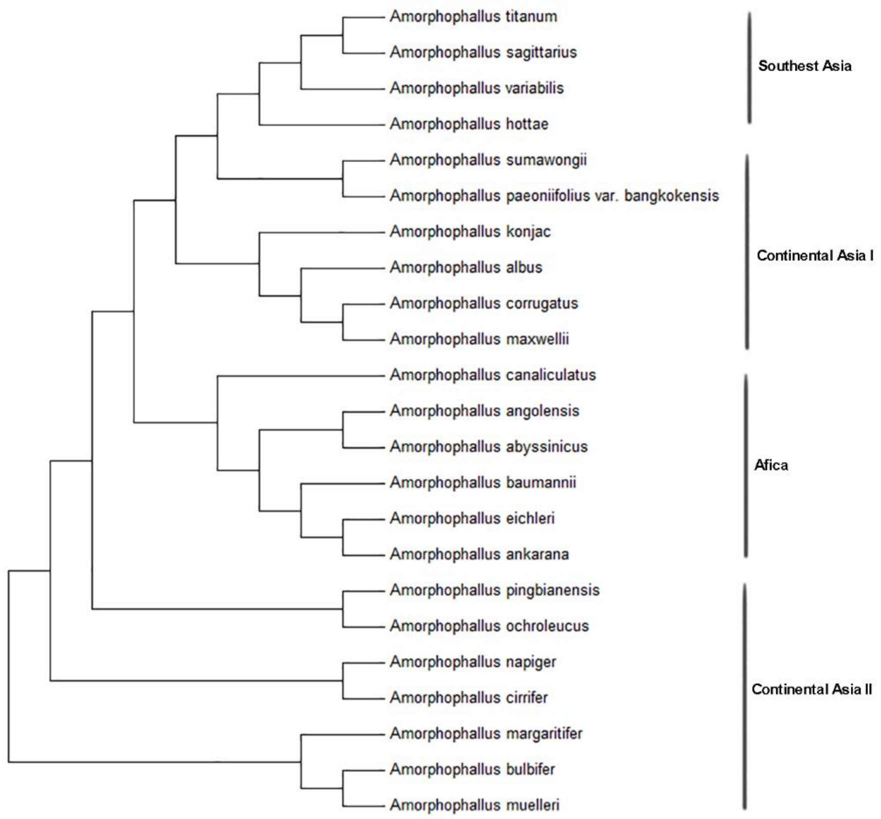

Figure S4 Phylogenetic tree based on rbcL gene sequences from our four Amorphophallus species and other Amorphophallus genus.

Table S1 Cp genomes of other species for phylogenetic analysis

Species	Accession	Genome size （bp）
Hordeum vulgare	NC_008590	136462
Oryza sativa	X15901	134525
Panicum virgatum	NC_015990	139619
Saccharum hybrid	NC_005878	141182
Sorghum bicolor	NC_008602	140754
Triticum aestivum	NC_002762	134545
Zea mays Maize	NC_001666	140384
Acorus gramineus	NC_026299	152849
Colocasia esculenta	NC_016753	162424
Allium cepa	NC_024813	153538
Dieffenbachia seguine	NC_027272	163699
Glycine max	NC_007942	152218
Phaseolus vulgaris	NC_009259	150285
Vigna radiata	NC_013843	151271
Solanum tuberosum	DQ231562	155312
Solanum lycopersicum	NC_007898	155461
Nicotiana tabacum	Z00044	155943
Capsicum annuum	NC_018552	156781
Raphanus sativus	NC_024469	153368

Brassica napus	NC_016734	152860
Zingiber spectabile	NC_020363	155890
Cathaya argyrophylla	NC_014589	107122
Cedrus deodara	NC_014575	119299
Cryptomeria japonica	NC_010548	131810
Podocarpus lambertii	NC_023805	133734
Populus alba	NC_008235	156505

Table S2 The *matK* informations of some Amorphophallus species for phylogenetic analysis

Species	Accession no.	Origin
Amorphophallus maxwellii	AF387405.1	Thailand, Kanchanaburi
Amorphophallus krausei	AF387399.1	Thailand, Khlong Lam Nai
Amorphophallus lewallei	AF387401.1	Burundi
Amorphophallus dracontioides	AF387389.1	Ghana, Legon Accra
Amorphophallus taurostigma	AF387420.1	Madagascar, Tulear
Amorphophallus julaihii	KY490472.1	Malaysia, Kota Samarahan
Amorphophallus eichleri	AF387391.1	Africa, cult (origin unknow)
Amorphophallus gigas	KY490484.1	Malaysia, Kota Samarahan
Amorphophallus titanum	KY490479.1	Indonesia, Sumatra
Amorphophallus variabilis	KY490475.1	Java, Kebun, Raya
Amorphophallus hewitii	KY490449.1	East Malaysia, Sarawak
Amorphophallus boyceanus	KY490478.1	Malaysia, Kota Samarahan
Amorphophallus eburneus	KY490458.1	Malaysia, Sarawak
Amorphophallus angulatus	KY490473.1	Malaysia, Kota Samarahan
Amorphophallus zenkeri	AF387424.1	Cameroom
Amorphophallus cirrifer	AF387386.1	Thailand, Saraburi
Amorphophallus atroviridis	KY490482.1	Malaysia, Kota Samarahan
Amorphophallus pingbianensis	AF387412.1	China, Yunan

Table S3 The *rbcL* informations of some Amorphophallus species for phylogenetic analysis

Species	Accession no.	Origin
Amorphophallus sumawongii	AF497099.1	Thailand, Ban Boa Nan Ching
Amorphophallus napiger	AF497089.1	Thailand, Phitsanulok
Amorphophallus pingbianensis	AF497093.1	China, Yunan
Amorphophallus baumannii	AF497063.1	Ghanh, Brong-Ahafo
Amorphophallus corrugatus	AF497070.1	Thailand, Chiang Mai
Amorphophallus cirrifer	AF497067.1	Thailand, Saraburi
Amorphophallus angolensis	AF497061.1	Gabon
Amorphophallus maxwellii	AF497086.1	Thailand, Kanchanaburi
Amorphophallus hottae	AF497079.1	Malaysia, Sarawak
Amorphophallus margaritifer	AF497085.1	India, Bengal
Amorphophallus canaliculatus	AF497066.1	Gabon
Amorphophallus titanum	AF497102.1	Indonesia, Sumatra
Amorphophallus sagittarius	AF497097.1	Indonesia, Java

Amorphophallus paeoniifolius var. bangkokensis	DQ012500.1	Indonesia, Sumatra
Amorphophallus ankarana	AF497062.1	Madagascar
Amorphophallus abyssinicus	AF497060.1	Nigeria
